# Mesenchymal Stromal Cells for Antineoplastic Drug Loading and Delivery

**DOI:** 10.3390/medicines4040087

**Published:** 2017-11-23

**Authors:** Francesco Petrella, Isabella Rimoldi, Stefania Rizzo, Lorenzo Spaggiari

**Affiliations:** 1 Department of Thoracic Surgery, European Institute of Oncology, 20141 Milan, Italy; lorenzo.spaggiari@ieo.it; 2 Department of Oncology and Hemato-oncology, University of Milan, 20141 Milan, Italy; 3 Department of Farmaceutical Sciences, University of Milan, 20141 Milan, Italy; isabella.rimoldi@unimi.it; 4 Department of Radiology, European Institute of Oncology, 20141 Milan, Italy; stefania.rizzo@ieo.it

**Keywords:** drug loading, drug delivery, mesenchymal stromal cell, paclitaxel

## Abstract

Mesenchymal stromal cells are a population of undifferentiated multipotent adult cells possessing extensive self-renewal properties and the potential to differentiate into a variety of mesenchymal lineage cells. They express broad anti-inflammatory and immunomodulatory activity on the immune system and after transplantation can interact with the surrounding microenvironment, promoting tissue healing and regeneration. For this reason, mesenchymal stromal cells have been widely used in regenerative medicine, both in preclinical and clinical settings. Another clinical application of mesenchymal stromal cells is the targeted delivery of chemotherapeutic agents to neoplastic cells, maximizing the cytotoxic activity against cancer cells and minimizing collateral damage to non-neoplastic tissues. Mesenchymal stem cells are home to the stroma of several primary and metastatic neoplasms and hence can be used as vectors for targeted delivery of antineoplastic drugs to the tumour microenvironment, thereby reducing systemic toxicity and maximizing antitumour effects. Paclitaxel and gemcitabine are the chemotherapeutic drugs best loaded by mesenchymal stromal cells and delivered to neoplastic cells, whereas other agents, like pemetrexed, are not internalized by mesenchymal stromal cells and therefore are not suitable for advanced antineoplastic therapy. This review focuses on the state of the art of advanced antineoplastic cell therapy and its future perspectives, emphasizing in vitro and in vivo preclinical results and future clinical applications.

## 1. Introduction

The ultimate goal of cancer chemotherapy is to optimize patient outcomes by increasing the drug concentration in the target tissues, thereby enhancing therapeutic efficacy, while simultaneously decreasing the exposure of healthy cells and tissues to reduce toxicity. Nanomedicines shift the tissue distribution of chemotherapeutic drugs, thereby significantly reducing the dose-limiting adverse effects while maintaining or even improving their efficacy [1].

Synthetic lipid- or polymer-based carrier systems or natural carriers like extracellular vesicles, viruses, bacteria and cells have been employed as drug carriers [2]. Mesenchymal stromal cells (MSC) are a population of undifferentiated multipotent adult cells possessing extensive self-renewal properties and the potential to differentiate into a variety of mesenchymal lineage cells [3,4]. They express broad anti-inflammatory and immunomodulatory activity on the immune system and after transplantation can interact with the surrounding microenvironment, promoting tissue healing and regeneration. For this reason, MSC have been widely used in the field of regenerative medicine, both in preclinical and clinical settings [5,6]. More recently, they have been advocated as natural carriers for targeted delivery of chemotherapeutic agents to neoplastic cells, maximizing the cytotoxic activity against cancer cells, and minimizing collateral damage to normal tissues [7]. In the present review, we describe MSC drug loading and delivery as potential new tool in clinical practice for oncologists, with specific regard to the most used chemotherapeutic agents like paclitaxel, gemcitabine, and pemetrexed.

## 2. Mesenchymal Stromal Cells

MSC are undifferentiated multipotent adult cells defined as plastic-adherent, fibroblast-like cells possessing extensive self-renewal properties and the in vivo and in vitro potential to differentiate into osteogenic, chondrogenic, and adipogenic lineages when cultured in specific inducing media [3]. MSC have an immune phenotype evading the host immune system, thus allowing allogenic transplantation without immunosuppression [8]. After transplantation into host tissues, MSC can interact with the surrounding microenvironment, stimulating tissue healing and regeneration by “cross talking” with other cells within the damaged tissue [9].

Initially discovered in bone marrow, MSC can be isolated from a wide spectrum of adult and foetal tissues like umbilical cord, adipose tissue, periosteum, tendon, dental pulp, cornea, thymus, spleen, brain, liver, placenta, and synovial and amniotic fluids [3]. Besides their potential to differentiate into adipocytes, osteoblasts, and chondroplasts, MSC can also differentiate into other mesodermal, endodermal, and ectodermal lineages, such as cardiomyocytes, skeletal myocytes, endothelial cells, tenocytes and hepatocytes, neuronal cells, photoreceptor cells, insulin-producing cells, epidermal and sebaceous duct cells, and renal tubular epithelial cells [10]. MSC are also able to migrate to sites of injury and engraftment, responding to chemokines, cytokines, and growth factors [11] and exerting local reparative effects mainly via the paracrine secretion of anti-inflammatory and wound-healing soluble factors [12].

**Figure 1:**
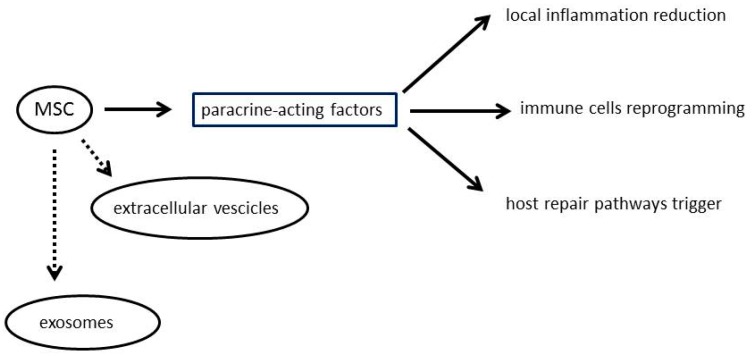
Potential mechanisms of mesenchymal stromal cells (MSC) actions.

Thanks to these characteristics, MSC are being exploited as an experimental therapy for a wide spectrum of human diseases. Current knowledge indicates that MSC effectively impact on disease via the secretion of paracrine-acting factors to reduce local inflammation, reprogramme immune cells, and trigger host repair pathways. It was recently discovered that MSC also produce extracellular vesicles—including exosomes—carrying as cargo mRNAs, microRNAs, and proteins inducing non-autonomous therapeutic changes within the damaged host tissue [13] (Figure 1).

## 3. Drug Loading and Drug Delivery by Mesenchymal Stem Cells

New therapeutic approaches to the cell-based delivery of chemotherapeutic drugs have been widely explored thanks to the capacity of MSC to migrate and engraft into tumours after intravenous administration [14]. After exposure to high doses of chemotherapeutic drugs like paclitaxel, MSC have been shown to accumulate intracellularly and deliver the antineoplastic agents without any genetic modifications, thereby decreasing tumour proliferation [15].

Many different methods of drug delivery have been described in the last decade, including immunoconjugates for targeting tumour-specific antigens, nanoparticles, and genetically modified stem cells; glycoengineering protocols to induce expression of non-natural azide groups on the surface of MSCs without altering their viability or tumour homing capacities have been reported, as well as nano-engineered MSCs were prepared by treating human MSCs with drug-loaded polymeric nanoparticles [16,17,18,19].

However, non-modified MSC are probably the best choice for anticancer drug delivery as they readily adapt to culture conditions and home to pathological tissues when injected in vivo and possess intrinsic antineoplastic activity [15]. This technique, however, is still experimental; only experiments on cellular lines or small animals have been performed until now, although with very promising results. For an experienced biologist, the procedure is quite easy, and it is not time consuming or expensive.

On the one hand, MSC hold great promise for oncology because they release active soluble factors and play an effective immunomodulatory role. They can also cross the blood brain barrier, thus representing a potential therapeutic tool for adult and paediatric brain tumours [20,21]. On the other, the issue of whether MSC cross-talk with the tumour microenvironment boosts tumour suppression or instead favours tumour growth remains unsettled [22]. For this reason, further experimental and preclinical studies are needed before switching MSC application as drug carriers to clinical practice. In fact, due to international regulatory dispositions and mainly to the lack of sufficient preclinical data, no clinical application has been performed until now.

Several hypotheses have been put forward to explain MSC antineoplastic activity, including inhibition of proliferation-related signalling pathways, angiogenesis suppression, and cell cycle inhibition [23,24,25].

Paclitaxel and gemcitabine are the chemotherapeutic drugs best loaded by mesenchymal stromal cells and delivered to neoplastic cells. Although pemetrexed has shown promising results in vivo for malignant mesothelioma therapy, the drug is not internalized by MSC and hence cannot benefit from this method for the time being.

The three lead compounds currently used in anticancer therapy, paclitaxel, gemcitabine and pemetrexed, have shown a wide range of activity focusing on different targets and still represent the first-line chemotherapy, especially against solid tumours.

## 4. Paclitaxel

**Figure 2:**
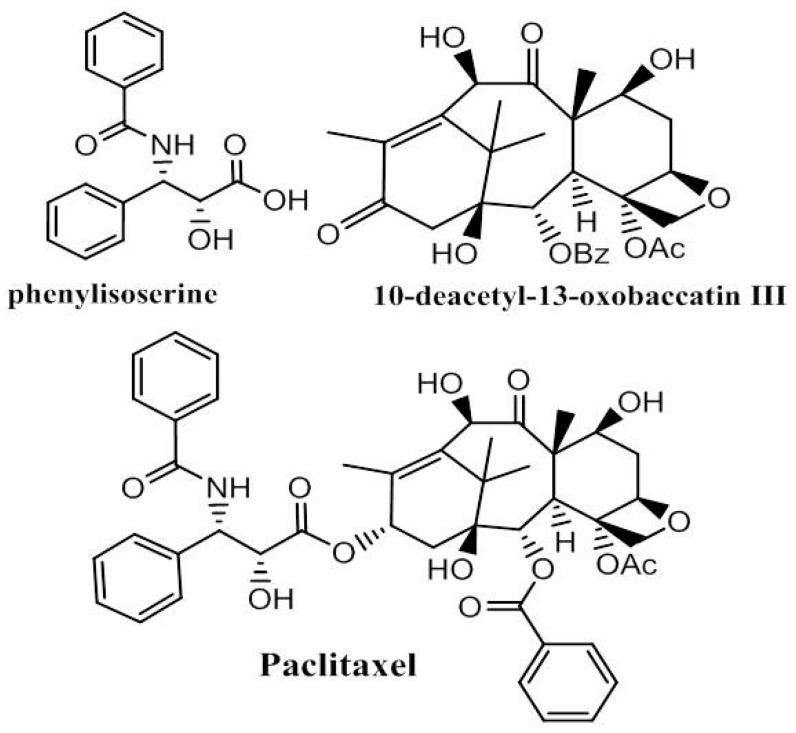
Paclitaxel obtained by a coupling reaction between (2*R*,3*S*)-*N*-benzoyl-3-phenylisoserine and the baccatin III core structure.

Among the plant-derived drugs used in the treatment of an enormous array of pathologies, paclitaxel (Figure 2) plays a significant role in cancer therapy, eliciting its activity by binding to the β-tubulin subunits in microtubules and influencing the depolarized/polarized equilibrium. The discovery of this antineoplastic agent from *Taxus brevifolia* [26] inspired the search for new taxoids chemically extracted from plant components. Paclitaxel is characterized by a chiral lateral chain (N-benzoyl-phenyl-isoserine group) and a taxoid ring. Both groups are necessary for the drug’s biological activity. The different synthetic approaches studied involve semi-synthesis in which the chiral lateral chain, obtained through bio- or organometallic catalysis [27,28,29,30], reacts with the baccatin III core structure isolated by *Taxus* species [31,32,33,34] (Figure 2).

## 5. Gemcitabine

**Figure 3:**
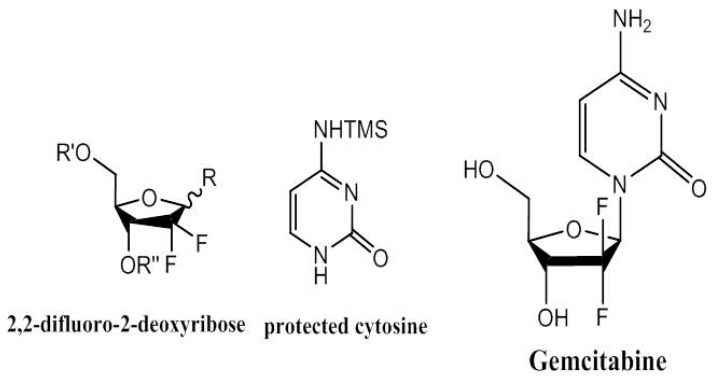
Gemcitabine obtained by a combination of protected and activated pentose and a substituted cytosine base.

Gemcitabine [35] (Figure 3) is a nucleoside analogue formed by a deoxy-difluorinated d-ribose in combination with a pyrimidine base (cytosine). The drug’s activity relies on the inhibition of ribonucleotide reductase and DNA synthesis against different types of solid tumours. Many gemcitabine synthesis strategies have been employed, especially those building enantioenriched nucleosides starting from natural sugars. This strategy bypasses the need for anomeric activation at the oxygen atom by synthesis of enantioselective prefabricated building blocks in which an appropriate leaving group, necessary during the coupling reaction with the nucleobase, is selectively introduced in the de novo synthetic sequence [36,37,38,39]. Other approaches also focused on modifying either the substituted nucleoside or the nucleobase [40,41,42].

## 6. Pemetrexed

**Figure 4:**
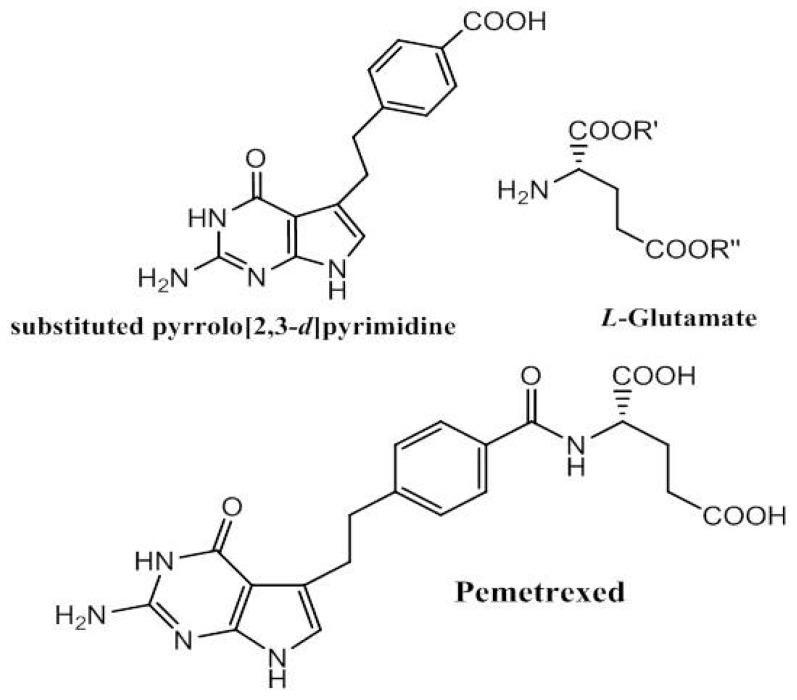
Pemetrexed synthetic pathway: 2,6-diamino-4(3*H*)-pyrimidinone as starting material to obtain substituted pyrrolo [2,3-*d*] pyrimidine, which reacts in a conventional peptide coupling with glutamate.

Pemetrexed (Figure 4) is a multi-target inhibitor of folate-dependent enzymes and plays a crucial role in blocking DNA and RNA replication by nucleobase biosynthesis. The folate metabolism inhibitors have antineoplastic activity especially in treating haematologic and solid tumours [43,44]. The synthetic pathway was mainly developed to enhance the efficiency and yield of the total synthesis starting from 2,6-diamino-4(3*H*)-pyrimidinone through different condensation steps to the last peptide coupling with chiral glutamate [45,46,47,48]. Different structure-activity relationship modifications have been implemented either on substituted pyrrolo [2,3-*d*] pyrimidine [49,50,51] or in the bridge between this substance and the benzoyl ring in the side chain [52].

## 7. Other Drugs Potentially Deliverable by MSC

Although paclitaxel and gemcitabine are the most common antineoplastic drugs potentially deliverable by MSC, in the last decade, several other toxic compounds have been experimentally tested.

Kosaka et al. reported that MSCs expressing cytosine deaminase and concurrent 5-fluorocytosine administration could improve the survival of rats bearing 9 L gliomas [53]. Ryu et al. demonstrated that MSCs loaded with herpes simplex virus type I thymidine kinase may increase the survival of glioma-bearing mice [54]. Li et al. showed that silica nanorattle-doxorubicin particles could be anchored to MSCs in a system called “nanoparticulate patches” [55]. Loaded cells could migrate towards U251 cancer cells both in vitro and in vivo. Roger et al. demonstrated that marrow-isolated adult multilineage inducible cells (MIAMI cells) containing ferrociphenol could induce cytotoxicity in U87MG glioma cells in vitro via a transwell system assay [56]. In a report from Rachakatla et al., neural progenitor cells were loaded with magnetic nanoparticles and delivered to mice suffering from malignant melanoma. Using an alternating magnetic field, hyperthermia was induced, and significant tumor decrease was observed [57].

Present results combining stem cells and nanoparticles for the induction of toxicity toward neoplastic cells demonstrate a promising proof-of-concept, but more work needs to be developed to confirm that stem cells effectively improve the efficacy of free-standing nanoparticle systems.

## 8. Clinical Perspectives

Pancreatic ductal adenocarcinoma (PDAC) is the most common type of pancreatic cancer, being the fourth leading cause of cancer death in the United States [58]. PDAC resistance to radiotherapy and chemotherapy represents a major limit to the efficacy of these treatments, resulting in an extremely disappointing overall five-year survival rate (about 5%) [59]. The current standard first-line treatment for locally advanced and metastatic PDAC is gemcitabine (GCB) and 5-fluorouracil, although a clinical response is obtained in only 10% of cases, so that novel therapies are urgently needed [60]. It has been experimentally demonstrated that GCB-loaded MSC can integrate into the tumour mass and deliver much higher concentrations of the chemotherapeutic drug than intravenous injection, thus acting as a “Trojan horse” for drug delivery [14].

Glioblastoma—also known as glioblastoma multiforme (GBM)—is the most aggressive brain cancer, representing 15% of brain tumours [60]. Treatment typically involves surgery followed by chemotherapy and radiation, but despite maximum treatment GBM usually recurs, the most common length of survival following diagnosis being 12 to 15 months with less than 3% to 5% of people surviving longer than five years [61]. MSC can migrate to and colonize GBM tumour xenografts when administered systemically or injected directly into the brain [22]. The MSC-tumour cell interaction resulted in significantly longer animal survival, reduced tumour volume, and impaired cell proliferation and vascularization. These preclinical results support the possible clinical use of MSC to treat GBM [22].

Melanoma—also known as malignant melanoma (MM)—is a malignant tumour developing from melanocytes of the skin or other tissues. Surgery plays a curative role for local disease, whereas immunotherapy, biologic therapy, radiation, or chemotherapy may improve survival for metastatic patients, although five-year survival rates drop from 98% among patients localized disease to 17% among those in whom spread has occurred. Paclitaxel-loaded MSC (PTX-MSC) have effectively inhibited lung metastasis formation in a murine B16 melanoma model, thus representing a potential therapeutic model for MM lung metastases [62].

Multiple myeloma is a neoplasm suppressing osteoblastogenesis by bone marrow mesenchymal stromal cells (BM-MSC). The role of MSC for multiple myeloma treatment is widely debated because of the contradictory results on MSC’s ability to inhibit or stimulate cancer growth. MSC could serve as vehicles for targeted delivery of anti-tumour agents into bone marrow. It has been experimentally demonstrated that PTX-loaded BM-MSC are active on the proliferation of a human myeloma cell line, expressing an intense suppression of myeloma cell growth. This suggests that drug-loaded MSC could represent an effective method to deliver the chemotherapeutic agent into the bone marrow [63].

Malignant pleural mesothelioma (MPM) is a cancer related to asbestos exposure whose incidence is expected to peak in 2020–2025 in Europe and Japan. The current standard first-line treatment is a platinum-based doublet containing a third-generation antifolate like pemetrexed (PMX) or ralitrexed. There are no approved second-line treatments for MPM as its prognosis is extremely poor, making it a disease setting to test new drugs. Initial experimental observations showed that PMX is not internalized and released by MSC. However, based on previous results with PTX-primed MSC, further experimental studies demonstrated that PTX-loaded MSC showed an excellent capacity to inhibit MPM cell proliferation, hitherto representing the best in vitro combination (drug + cells) for MPM therapy [7,64] (Table 1).

## 9. Conclusions

MSC antineoplastic drug loading and drug delivery represents a promising new field of chemotherapy and advanced cell therapy for oncological diseases, in particular those with a poor prognosis. Successful experimental tests on models of pancreatic adenocarcinoma, glioblastoma, melanoma, multiple myeloma and malignant pleural mesothelioma have yielded very encouraging in vitro results. Phase I clinical studies are now required for further confirmation of the feasibility and efficacy of advanced cell therapy. Adipose-derived MSC as well as nanoparticles may represent—in the future—the new frontiers of drug loading and delivery.

## Figures and Tables

**Table 1:** Possible oncologic indications for clinical use of MSC.

Tumor	Standard Care	MSC Action
Pancreatic ductal adenocarcinoma	Gemcitabine and 5-fluorouracil	“Trojan horse”
Glioblastoma multiforme	Surgery followed by chemotherapy and radiation	Reduction of tumour volume, impairment of cell proliferation and vascularization.
Malignant melanoma	Surgery and immunotherapy or biologic therapy and radiation or chemotherapy	Inhibition of lung metastasis in a murine melanoma model
Multiple myeloma	Chemotherapy	Intense suppression of myeloma cell growth
Malignant mesothelioma	Platinum-based doublet containing a third-generation antifolate + surgery + radiotherapy	PTX-loaded MSC strongly inhibit MPM cell proliferation

## Abbreviations

MSC	mesenchymal stromal cells
PDAC	pancreatic ductal adenocarcinoma
GCB	gemcitabine
GBM	glioblastoma multiforme
PTX	paclitaxel
BM	bone marrow
MPM	malignant pleural mesothelioma
PMX	pemetrexed
